# Quadruplex Droplet Digital PCR Assay for Screening and Quantification of SARS-CoV-2

**DOI:** 10.3390/ijms25158157

**Published:** 2024-07-26

**Authors:** Rong Li, Zaobing Zhu, Yongkun Guo, Litao Yang

**Affiliations:** 1 Joint International Research Laboratory of Metabolic and Developmental Sciences, School of Life Sciences and Biotechnology, Shanghai Jiao Tong University, Shanghai 200240, China; lirong19901102@sjtu.edu.cn (R.L.); guoyongkun@sjtu.edu.cn (Y.G.); 2 Department of Neurosurgery, Shanghai Changhai Hospital, Naval Medical University, Shanghai 200433, China; 3 Institute of Plant Protection, Jiangsu Academy of Agricultural Sciences, Nanjing 210014, China; zbzhu152@sjtu.edu.cn; 4 Yazhou Bay Institute of Deepsea Sci-Tech, Shanghai Jiao Tong University, Sanya 572025, China

**Keywords:** SARS-CoV-2, droplet digital PCR, quadruplex assay

## Abstract

The ongoing COVID-19 pandemic, caused by the rapid global spread of severe acute respiratory syndrome coronavirus 2 (SARS-CoV-2) since early 2020, has highlighted the need for sensitive and reliable diagnostic methods. Droplet digital PCR (ddPCR) has demonstrated superior performance over the gold-standard reverse transcription PCR (RT-PCR) in detecting SARS-CoV-2. In this study, we explored the development of a multiplex ddPCR assay that enables sensitive quantification of SARS-CoV-2, which could be utilized for antiviral screening and the monitoring of COVID-19 patients. We designed a quadruplex ddPCR assay targeting four SARS-CoV-2 genes and evaluated its performance in terms of specificity, sensitivity, linearity, reproducibility, and precision using a two-color ddPCR detection system. The results showed that the quadruplex assay had comparable limits of detection and accuracy to the simplex ddPCR assays. Importantly, the quadruplex assay demonstrated significantly improved performance for samples with low viral loads and ambiguous results compared to the standard qRT-PCR approach. The developed multiplex ddPCR represents a valuable alternative and complementary tool for the diagnosis of SARS-CoV-2 and potentially other pathogens in various application scenarios beyond the current COVID-19 pandemic. The improved sensitivity and reliability of this assay could contribute to more effective disease monitoring and antiviral screening during the ongoing public health crisis.

## 1. Introduction

The COVID-19 pandemic, caused by the SARS-CoV-2 virus, has rapidly spread worldwide, with millions of confirmed cases reported to date. Despite the development of drugs, vaccines, and other interventions, a definitive solution to control this pandemic remains elusive. Consequently, the ability to accurately detect, isolate, and treat infected individuals remains crucial for clinical diagnostics and disease management [1,2].

The gold standard for SARS-CoV-2 diagnosis is the quantitative reverse transcription polymerase chain reaction (qRT-PCR) assay, which is widely implemented globally. However, qRT-PCR has several limitations, including the need for a standard curve to perform relative quantification, which can introduce variation due to differences in amplification efficiency between samples and standards [3]. Additionally, the accurate detection of low- and very low-positive samples remains challenging [4].

Furthermore, it has been reported that different primer–probe sets targeting the SARS-CoV-2 genome can lead to inconclusive results and a significant number of false-negative reports [5]. This can adversely impact timely diagnosis, early treatment, prevention of transmission, and assessment of discharge criteria.

Droplet digital PCR (ddPCR) has emerged as an attractive alternative to qRT-PCR for the direct quantification of nucleic acids [6]. Numerous studies have highlighted the superior analytical sensitivity and better reproducibility of ddPCR compared to qRT-PCR for the detection of SARS-CoV-2 [5,7,8,9,10,11,12]. Importantly, ddPCR has been shown to reliably detect SARS-CoV-2 in clinical samples that tested negative by qRT-PCR, thereby reducing the risk of false-negative results [5]. Moreover, ddPCR offers the advantage of multiplexing, which can improve the sensitivity of the assay, reduce costs, and increase the chances of target detection [13,14]. Additionally, the concentration of target molecules in smaller reactions can mitigate the bias introduced by PCR inhibitors often found in clinical specimens [14,15].

In this study, we developed a quadruplex ddPCR assay for the efficient screening and quantification of SARS-CoV-2. The assay targets four viral genomic regions: the envelope (E) gene, the nucleocapsid (N) gene, the RNA-dependent RNA polymerase (RdRp) gene, and an open reading frame 1 a/b (ORF1 a/b) gene. We evaluated the sensitivity, dynamic range, and performance of this quadruplex ddPCR assay using simulated practical sample detection.

## 2. Results

### 2.1. Development and Optimization of a Quadruplex ddPCR Assay for SARS-CoV-2 Detection

**Figure 1:**
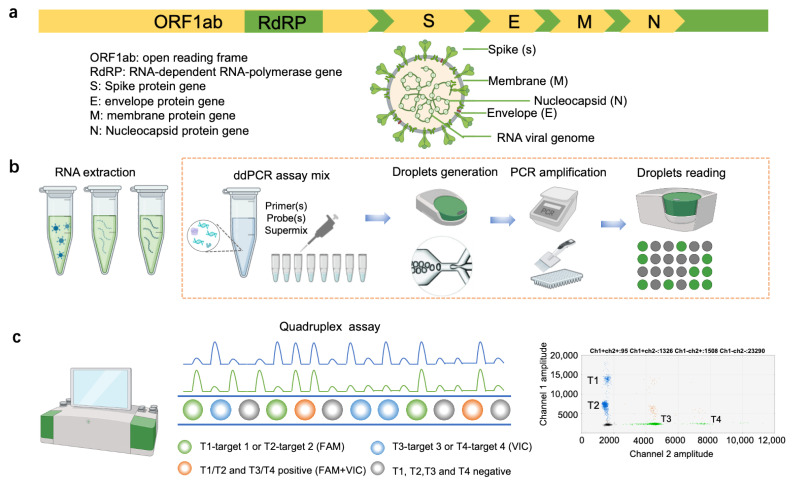
Principle and workflow of the quadruplex ddPCR assay for SARS-CoV-2 detection: (**a**) Schematic representation of the SARS-CoV-2 genome organization. (**b**) Sample handling, preparation, and ddPCR workflow. (**c**) Data analysis and droplet separation.

We developed a multiplex droplet digital PCR (ddPCR) test to detect SARS-CoV-2 by targeting various stable regions of the virus’s genome, as shown in Figure 1a. These regions include the open reading frame 1 ab (ORF1 ab), RNA-dependent RNA polymerase (RdRp), and key structural proteins such as the envelope (E), membrane (M), and nucleocapsid (N). Establishing a quadruplex ddPCR system typically involves optimizing and preparing the ddPCR reaction mixture, generating droplets, conducting RT-PCR amplification, and reading the droplet signals (Figure 1b). The ddPCR system used in this study features two channels capable of detecting four specific targets under varying concentrations of primers and probes. We designed a quadruplex assay that enhances multiplexing capabilities using non-interfering duplex reactions to simultaneously detect four SARS-CoV-2 markers. Targets T1 and T2 are assessed in channel 1 (FAM) and targets T3 and T4 in channel 2 (HEX/VIC). Each channel optimizes the intensity of the fluorescent signals emitted from each target with different concentrations of primer/probe sets (Figure 1c). Targets that test positive are subsequently identified and categorized based on the fluorescence intensity exhibited by the droplets in each channel.

### 2.2. Optimization and Development of a Quadruplex ddPCR Assay

The quadruplex ddPCR assay was optimized with different ratios of primers and probes. The optimized assay consisted of 10 μL of 2× ddPCR Supermix, each primer at 500 nM, the N and ORF1 ab gene probes at 250 nM, the E and RdRp gene probes at 500 nM, and a 1 μL template, made up to 20 μL with nuclease-free water. In the quadruplex ddPCR assay, both the ORF1 ab and RDRP targets were labeled with FAM fluorescence and read in channel 1. The N gene and E gene were labeled with VIC fluorescence in channel 2. In the FAM channel, ORF1 ab probes were added at a concentration of 0.5×, while RdRp probes were added at a 1× concentration (Supplementary Figure S1a,b). For the VIC channel, N probes were added at 0.5× concentration, while E probes were added at 1× concentration (Supplementary Figure S1c,d). The annealing temperature was also optimized, and 57 °C was found to be suitable for clear separation of the droplet clusters (Supplementary Figure S1e,f).

### 2.3. Analytical Performance of the Quadruplex ddPCR Assay

The specificity of the developed quadruplex ddPCR assay was evaluated, and a clear fluorescence signal was observed only in the reaction with SARS-CoV-2, while no positive droplet signal occurred for non-target samples, as shown in Table 1. Seven different respiratory disease viruses were used as templates. All the specificity tests of the simplex and quadruplex ddPCR were performed in triplicate, confirming the high specificity of the developed quadruplex ddPCR assays for SARS-CoV-2.

The limit of detection (LOD) and limit of quantification (LOQ) of the quadruplex ddPCR assay were determined using gradient DNA dilutions with concentrations corresponding to 20,000, 4000, 800, 160, 32, 6.4, and 1.28 copies per reaction as templates. Positive droplet signals were observed in the reactions with all the gradient dilutions except for the reaction with the dilution of 1.28 copies per reaction (Table 2). Therefore, the LOD and LOQ of the quadruplex assay were determined to be around 6.4 copies per reaction, demonstrating the higher sensitivity of the quadruplex ddPCR assay compared to the qRT-PCR method (around 20 copies per reaction) and multiplex qRT-PCR (around 20 copies per reaction) used in routine analysis [4,14].

**Figure 2:**
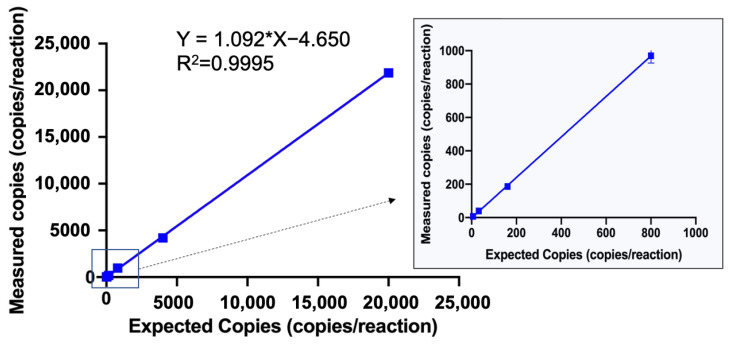
Dynamic range and correlation of the quadruplex ddPCR assay for SARS-CoV-2 quantification. The dynamic range of the quadruplex ddPCR assay was determined using a series of gradient DNA dilutions with 20,000, 4000, 800, 160, 32, and 6.4 copies per reaction.

The dynamic range of the quadruplex ddPCR assay was determined using a series of gradient dilutions with the expected concentrations of 20,000, 4000, 800, 160, 32, and 6.4 copies per reaction. Reactions within the range of 20,000 to 6.4 copies per reaction were successfully quantified with a relative standard deviation (RSD) ≤ 25%, and a good linear relationship with an R^2^ value of 0.9999 was calculated between the expected copies and measured copies (Figure 2). These results demonstrated that the established quadruplex ddPCR assay had an effective dynamic range between 20,000 copies and 6.4 copies per reaction.

The quadruplex ddPCR reactions demonstrated great repeatability within the range of 20,000 to 6.4 copies per reaction, with RSD values from all nine replicates being ≤24% (Table 3), indicating consistent results across multiple tests.

### 2.4. Comparison of Quadruplex and Simplex ddPCR for SARS-CoV-2 Quantification

**Figure 3:**
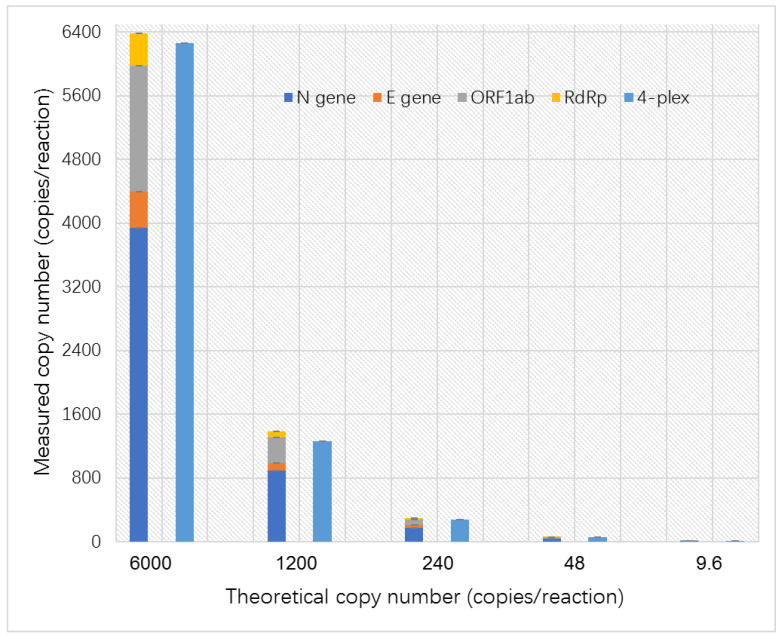
The precision quantification analysis of the quadruplex ddPCR assay employing simulated SARS-CoV-2 samples as examples.

The performance of the quadruplex ddPCR assay was evaluated by comparing its SARS-CoV-2 quantification results with those of simplex ddPCR analysis. In the simplex ddPCR analysis, each sample was tested individually in four simplex ddPCR assays, and the copy number of each sample was calculated as the sum of the quantified results from four simplex ddPCR assays. However, the quantified results could be obtained in one quadruplex ddPCR reaction directly. The copy numbers of all target genes (N, E, ORF1 ab, and RdRp) were comparable between the two methods for samples with different amounts of target genes (6000, 1200, 240, 48, and 9.6 copies) (Figure 3, Table 4). For example, a total of 6384 copies of the sample with a theoretical value of 6000 copies was quantified in simplex ddPCR analysis, and 6262 copies were obtained in quadruplex ddPCR. In particular, different results were observed for the samples with 9.6 copies, where negative results were obtained in simplex ddPCR analysis, while positive results with copy numbers of 10.2 were obtained in quadruplex ddPCR analysis. The quadruplex ddPCR assay exhibited high accuracy and sensitivity, even detecting positive results in the sample with 9.6 copies where simplex ddPCR analysis was negative. In addition, we observed that the quantified results of simplex ddPCR analysis were always slightly higher than those of quadruplex ddPCR due to measurement errors and the accumulation of systematic errors. Overall, the quadruplex ddPCR assay exhibited high accuracy and sensitivity compared to the simplex ddPCR analysis.

### 2.5. Screening and Quantification of Practical Samples

**Figure 4:**
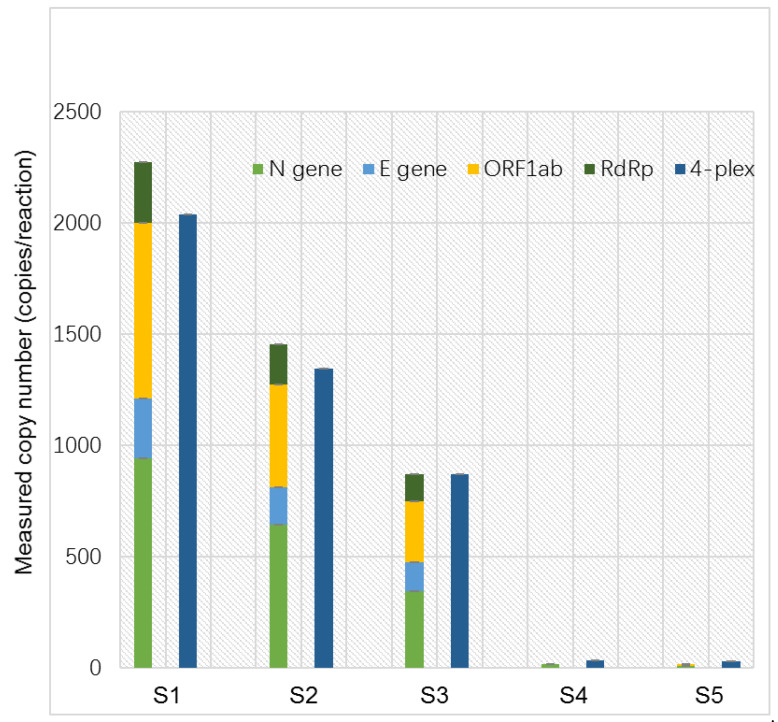
Screening and quantification of practical samples using both the simplex ddPCR and quadruplex ddPCR analyses.

To further evaluate the performance of the quadruplex ddPCR assay, thirteen clinical samples (S1–S13) were also tested using both simplex and quadruplex ddPCR assays. (Figure 4, Table 5). Positive signals were detected, and the copy numbers of the target genes were quantified in the positive samples (S1–S3) for all simplex and quadruplex ddPCR assays. Eight samples (S6–S13) showed negative results. In samples S4 and S5, positive signals were detected in the quadruplex ddPCR assay and partially in the simplex ddPCR assays (N gene in S4 and ORF1 ab gene in S5). The quantified results were significantly higher in the quadruplex ddPCR assay compared to the simplex ddPCR assays. This discrepancy might be due to the presence of trace amounts of the virus in S4 and S5, falling below the LOQ and LOD of the simplex ddPCR assays. The results from S4 and S5 indicate that the quadruplex ddPCR assay demonstrates better accuracy and sensitivity than the simplex ddPCR assays. Meanwhile, we discovered that both the simplex and quadruplex ddPCR assays demonstrated greater sensitivity compared to the qRT-PCR results for each sample provided by Jiangsu Bio-Perfectus Technologies Co., Ltd., Taizhou, China (Supplementary Table S3).

## 3. Discussion

As the SARS-CoV-2 pandemic and its genome continue to evolve rapidly, it is crucial to simultaneously screen multiple targets to confirm the presence of SARS-CoV-2 and avoid false-negative results during early, sensitive, and reliable diagnosis [16]. However, recent real-time RT-PCR (RT-qPCR) assays have reported insufficient sensitivity to identify all cases of SARS-CoV-2 infection, with up to 20% false-negative rates [17,18]. Therefore, it is essential to develop complementary laboratory assays for precise analysis.

In this study, we successfully established a novel quadruplex droplet digital PCR (ddPCR) assay by designing and optimizing primer/probe sets targeting four SARS-CoV-2 genes (N, E, ORF1 ab, and RdRp). The developed quadruplex ddPCR assay was thoroughly validated for specificity, dynamic range, sensitivity, repeatability, and precision. Our quadruplex ddPCR assay exhibited a wide dynamic range of 20,000 to 6.4 copies per reaction, with an absolute limit of detection (LOD) and limit of quantification (LOQ) of 6.4 copies. The assay demonstrated the ability to correctly identify weakly positive samples, making it suitable for diagnostic purposes.

The quadruplex ddPCR format allowed for the simultaneous amplification and quantification of all four targets in a single reaction, potentially improving the efficiency of SARS-CoV-2 preliminary screening compared to the simplex ddPCR method, especially during the early stages of viral replication or when the virus is present at lower levels. Furthermore, our results showed that the quadruplex ddPCR assay exhibited greater sensitivity than simplex ddPCR assays when tested with SARS-CoV-2 samples of varying concentrations.

The quadruplex ddPCR format reduced the overall experiment time and the amount of ddPCR reagents required, as all targets were tested in a single reaction. The ability of ddPCR to generate accurate quantitative data has had a significant impact on the study of viral agents of infectious diseases. Therefore, ddPCR should be considered as a complement to the gold-standard RT-qPCR-based diagnosis for the rapid identification of SARS-CoV-2 infection.

In conclusion, our research suggests that ddPCR holds promise as a molecular diagnostic tool for detecting low levels of the SARS-CoV-2 virus. As the demand for detection continues to grow, incorporating additional targets into the multiplex ddPCR assay for preliminary screening analysis may be essential, potentially leading to the integration of ddPCR into routine clinical practice. The quadruplex ddPCR assay developed in this study has the potential for further expansion and the addition of more targets for disease detection.

## 4. Materials and Methods

### 4.1. Materials

Certified reference materials (CRMs) for SARS-CoV-2 (GBW (E) 019089) and reference materials for 2019-nCoV pseudo virus RNA (NIM-RM5203) were obtained from the National Centre for Reference Materials (NCRM), Beijing, China. Six reference materials (RMs) for different respiratory viruses were kindly provided by the Shanghai Institute of Measurement and Testing Technology (SIMT), Shanghai, China. Details for all RMs are listed in Supplementary Table S1. Practical nasopharyngeal swab samples with known Ct values of q-RT-PCR were kindly supplied by Jiangsu BioPerfectus Technologies Co., Ltd., Taizhou, China, and used for further practical sample analysis. The SARS-CoV-2 RM RNA was extracted using QIAamp Viral RNA Kits (QIAGEN, Dusseldorf, Germany) and then reverse transcribed into cDNA using the FastKing RT Kit (TIANGEN, Beijing, China). The quality and quantity of extracted DNA or RNA were evaluated using a NanoDrop 2000 UV/vis spectrophotometer (NanoDrop Technologies, LLC, Wilmington, DE, USA).

### 4.2. Primers and Probes

China CDC primers and probes targeting the open reading frame 1 ab (ORF1 ab), envelope (E) gene regions, RNA-dependent RNA-polymerase gene (RdRp), and nucleoprotein (N) gene regions of SARS-CoV-2 were used to develop the simplex and multiplex ddPCR assays (Supplementary Table S2) [13]. The probes for the N gene and E gene were labeled with the FAM fluorophore, while the ORF1 ab gene and RdRp gene were labeled with the VIC fluorophore for optimized ddPCR. All primers and probes were provided by Thermo Fisher Biotech Company in Guangzhou, China.

### 4.3. Simplex ddPCR Assays

For the preparation and optimization of simplex ddPCR assays, each simplex ddPCR assay was composed of a primer and probe pair for a particular target and was carried out in a final volume of 20 μL. Each reaction contained 10 μL of 2× ddPCR Supermix for probes (No dUTP), 500 nM of forward and reverse primers for each target, 250 nM-500 nM of target probe, 1 μL of diluted cDNA, and nuclease-free water. Simplex ddPCR assays of each target were performed with an optimal concentration of primers and probes to maintain consistent fluorescence values of negative droplets. The procedure for simplex ddPCR assays was as follows: 5 min at 95 °C followed by 40 cycles of a two-step thermal profile comprising 30 s at 95 °C and 60 s at 58 °C at a ramp rate of 2.0 °C/s. After cycling, each sample was incubated at 98 °C for 10 min and then cooled to 4 °C.

### 4.4. Quadruplex ddPCR Assays

The quadruplex ddPCR assay, also known as the 4-plex ddPCR assay, was designed to detect four SARS-CoV-2 genetic targets (N, E, ORF1 ab, and RdRp) simultaneously. The assay consisted of four sets of primers and probes, with each channel (FAM and VIC) used to detect two targets at optimized primer and probe concentrations. The reaction mixture contained 10 μL of 2× ddPCR Supermix for Probes (No dUTP), primers at concentrations ranging from 250 nM to 1000 nM, probes at concentrations ranging from 250 nM to 500 nM, 1 μL of cDNA, and nuclease-free water to a final volume of 20 μL. The thermal cycling conditions for the 4-plex ddPCR assay were as follows: 5 min at 95 °C, followed by 40 cycles of 30 s at 95 °C and 60 s at 58 °C, with a ramp rate of 2.0 °C/s. After cycling, each sample was incubated at 98 °C for 10 min and cooled to 4 °C.

### 4.5. ddPCR Reactions and Data Analysis

The 20 μL reaction mixtures for simplex and multiplex ddPCR reactions were loaded into 8-well cartridges, and droplets were generated using the QX200 Droplet Generator (Bio-Rad, Pleasanton, CA, USA). The water-in-oil emulsions were then transferred to a 96-well plate and amplified in a T100 Thermal Cycler (Bio-Rad, Pleasanton, CA, USA). After PCR amplification, the plates were transferred to the QX200 Droplet Reader (Bio-Rad, Pleasanton, CA, USA), and data acquisition and analysis were performed using QuantaSoft software v1.7 (Bio-Rad, Pleasanton, CA, USA). Positive droplets containing amplification products were differentiated from negative droplets without amplification products by applying a fluorescence amplitude threshold. The data were further analyzed using Microsoft Excel 2019 spreadsheets. All simplex and multiplex ddPCR analyses were performed in triplicate.

### 4.6. Performance Evaluation of Quadruplex ddPCR Assay

The performance of the quadruplex ddPCR assay was evaluated for key parameters, including specificity, dynamic range, limit of detection (LOD), limit of quantification (LOQ), repeatability, precision, and applicability. A serial dilution of cDNA derived from the SARS-CoV-2 virus was used to assess the LOD and LOQ of the assay. The dynamic range of the quadruplex ddPCR was also determined. Additionally, the applicability of the 4-plex ddPCR assay was verified using practical samples.

## Figures and Tables

**Table 1:** The specificity of simplex and quadruplex ddPCR assays.

Respiratory Virus	N	E	ORF1 ab	RDRP	4-plex
COVID-19	+	+	+	+	+
Parainfluenza virus type 1	-	-	-	-	-
Influenza A virus (H1 N1)	-	-	-	-	-
Influenza B virus (Victoria)	-	-	-	-	-
SARS	-	-	-	-	-
MERS	-	-	-	-	-
NTC	-	-	-	-	-

**Table 2:** LOD and LOQ of the developed quadruplex assay.

Amount of Template	Copy Number/Reaction				SD	RSD (%)	Bias (%)
	1	2	3	avg cp			
20,000	21,820	21,720	22,080	21,873.3	185.83	0.85%	9.37%
4000	4680	3944	3978	4200.7	415.46	9.89%	5.02%
800	992	996	920	969.3	42.77	4.41%	21.17%
160	184	172	204	186.7	16.17	8.66%	16.67%
32	34	42	40	38.7	4.16	10.77%	20.83%
6.4	7.6	5.2	8.4	7.1	1.67	23.57%	10.42%
1.28	2.7	2.4	/	/	/	/	/

**Table 3:** The repeatability and reproducibility of the quadruplex assay.

Amount of Template	Repeat 1			Repeat 2			Repeat 3			avg cp	SD	RSD
	avg cp	SD	RSD	avg cp	SD	RSD	avg cp	SD	RSD			
20,000	20,840	731.2	3.51%	22,120	1250.5	5.65%	21,030	1189.2	5.65%	21,330.0	690.7	3.24%
4000	4458	337.8	7.58%	4918	834.8	16.97%	4494	646.1	14.38%	4623.3	255.8	5.53%
800	892	85.0	9.53%	762	99.4	13.04%	932	52.3	5.61%	862.0	88.9	10.31%
160	167	12.9	7.68%	180	14.4	8.01%	178	20.3	11.40%	175.1	6.8	3.89%
32	38	8.5	22.48%	42	7.0	16.67%	36	7.2	20.03%	38.7	3.1	7.90%
6.4	8.6	1.9	22.18%	5.9	0.9	15.93%	9.7	2.1	21.30%	8.1	1.9	23.98%
1.28	2.3	0.7	31.59%	2.2	0.6	27.27%	/	/	/	/	/	/

**Table 4:** The precision quantification results of the simplex and quadruplex ddPCR assays with different percentages.

Theoretical Copy Number	Simplex ddPCR									4-plex ddPCR		Bias Simplex to 4-plex
	N		E		ORF1 ab		RdRp		SUM			
	avg cp	RSD	avg cp	RSD	avg cp	RSD	avg cp	RSD	avg cp	avg cp	RSD	
6000	3940.0	3.66%	450.7	6.06%	1584.0	10.62%	409.3	4.27%	6384.0	6262.0	0.89%	1.91%
1200	893.3	8.48%	94.0	13.29%	320.0	14.54%	80.0	6.61%	1387.3	1258.7	10.01%	9.27%
240	173.7	16.65%	32.0	16.54%	72.7	15.16%	19.3	21.53%	297.7	279.3	5.96%	6.16%
48	35	18.74%	8.2	9.76%	12.1	19.07%	4.6	19.92%	59.9	57.0	12.30%	4.79%
9.6	8.2	19.96%	/	/	3.6	25.46%	/	/	11.8	10.2	18.00%	13.56%

**Table 5:** Quantification results from simplex and quadruplex ddPCR assays of the practical samples.

Theoretical Copy Number	Simplex ddPCR									4-plex ddPCR		Bias Simplex to 4-plex
	N		E		ORF1 ab		RDRP		SUM			
	avg cp	RSD	avg cp	RSD	avg cp	RSD	avg cp	RSD	avg cp	avg cp	RSD	
S1	940.0	5.63%	270.0	5.19%	790.7	9.11%	272.0	9.62%	2272.7	2038.7	3.78%	10.30%
S2	642.0	5.77%	169.3	8.30%	460.7	6.74%	180.0	4.01%	1452.0	1344.0	3.54%	7.44%
S3	343.7	3.40%	132.0	4.01%	272.7	2.58%	122.7	3.39%	871.0	833.3	4.58%	4.32%
S4	17.3	11.54%	/	/	/	/	/	/	17.3	32.7	3.55%	−89.02%
S5	11.3	12.85%	/	/	5.6	24.49%	/	/	16.9	31.2	5.13%	−84.62%
S6	/	/	/	/	/	/	/	/	/	/	/	/
S7	/	/	/	/	/	/	/	/	/	/	/	/
S8	/	/	/	/	/	/	/	/	/	/	/	/
S9	/	/	/	/	/	/	/	/	/	/	/	/
S10	/	/	/	/	/	/	/	/	/	/	/	/
S11	/	/	/	/	/	/	/	/	/	/	/	/
S12	/	/	/	/	/	/	/	/	/	/	/	/
S13	/	/	/	/	/	/	/	/	/	/	/	/

## Data Availability

Data is contained within the article and Supplementary Material.

## Supplementary Materials

The supporting information can be downloaded at: https://www.mdpi.com/article/10.3390/ijms25158157/s1.

## References

[1] Thirumugam G., Radhakrishnan Y., Ramamurthi S., Bhaskar J.P., Krishnaswamy B. (2023). A systematic review on impact of SARS-CoV-2 infection. Microbiol. Res..

[2] Nunnari G., Sanfilippo C., Castrogiovanni P., Imbesi R., Li Volti G., Barbagallo I., Musumeci G., Di Rosa M. (2020). Network perturbation analysis in human bronchial epithelial cells following SARS-CoV2 infection. Exp. Cell Res..

[3] Svec D., Tichopad A., Novosadova V., Pfaffl M.W., Kubista M. (2015). How good is a PCR efficiency estimate: Recommendations for precise and robust qPCR efficiency assessments. Biomol. Detect. Quantif..

[4] Vogels C.B.F., Brito A.F., Wyllie A.L., Fauver J.R., Ott I.M., Kalinich C.C., Petrone M.E., Casanovas-Massana A., Catherine Muenker M., Moore A.J. (2020). Analytical sensitivity and efficiency comparisons of SARS-CoV-2 RT-qPCR primer-probe sets. Nat. Microbiol..

[5] Suo T., Liu X., Feng J., Guo M., Hu W., Guo D., Ullah H., Yang Y., Zhang Q., Wang X. (2020). ddPCR: A more accurate tool for SARS-CoV-2 detection in low viral load specimens. Emerg. Microbes Infect..

[6] Aravind Kumar N., Aradhana S., Harleen Vishnuraj M.R. (2023). SARS-CoV-2 in digital era: Diagnostic techniques and importance of nucleic acid quantification with digital PCRs. Rev. Med. Virol..

[7] Liu X., Feng J., Zhang Q., Guo D., Zhang L., Suo T., Hu W., Guo M., Wang X., Huang Z. (2020). Analytical comparisons of SARS-COV-2 detection by qRT-PCR and ddPCR with multiple primer/probe sets. Emerg. Microbes Infect..

[8] Ma C., Sun Y., Huang Y., Gao Z., Huang Y., Pandey I., Jia C., Feng S., Zhao J. (2023). On-Chip Nucleic Acid Purification Followed by ddPCR for SARS-CoV-2 Detection. Biosensors.

[9] Jiang Y., Wang H., Hao S., Chen Y., He J., Liu Y., Chen L., Yu Y., Hua S. (2020). Digital PCR is a sensitive new technique for SARS-CoV-2 detection in clinical applications. Clin. Chim. Acta.

[10] Falzone L., Musso N., Gattuso G., Bongiorno D., Palermo C.I., Scalia G., Libra M., Stefani S. (2020). Sensitivity assessment of droplet digital PCR for SARS-CoV-2 detection. Int. J. Mol. Med..

[11] Martin A., Storto A., Le Hingrat Q., Collin G., André B., Mallory A., Dangla R., Descamps D., Visseaux B., Gossner O. (2021). High-sensitivity SARS-CoV-2 group testing by digital PCR among symptomatic patients in hospital settings. J. Clin. Virol..

[12] Burjanivova T., Lukacova E., Lucansky V., Samec M., Podlesniy P., Kolkova Z., Reizigova L., Grendar M., Turyova E., Holubekova V. (2023). Sensitive SARS-CoV-2 detection, air travel Covid-19 testing, variant determination and fast direct PCR detection, using ddPCR and RT-qPCR methods. Acta Virol..

[13] Cassinari K., Alessandri-Gradt E., Chambon P., Charbonnier F., Gracias S., Beaussire L., Alexandre K., Sarafan-Vasseur N., Houdayer C., Etienne M. (2021). Assessment of multiplex digital droplet RT-PCR as a diagnostic tool for SARS-CoV-2 detection in nasopharyngeal swabs and saliva samples. Clin. Chem..

[14] Morón-López S., Riveira-Muñoz E., Urrea V., Gutiérrez-Chamorro L., Ávila-Nieto C., Noguera-Julian M., Carrillo J., Mitjà O., Mateu L., Massanella M. (2023). Comparison of Reverse Transcription (RT)-Quantitative PCR and RT-Droplet Digital PCR for Detection of Genomic and Subgenomic SARS-CoV-2 RNA. Microbiol. Spectr..

[15] Taylor S.C., Laperriere G., Germain H. (2017). Droplet Digital PCR versus qPCR for gene expression analysis with low abundant targets: From variable nonsense to publication quality data. Sci. Rep..

[16] de Kock R., Baselmans M., Scharnhorst V., Deiman B. (2021). Sensitive detection and quantification of SARS-CoV-2 by multiplex droplet digital RT-PCR. Eur. J. Clin. Microbiol. Infect. Dis..

[17] Alteri C., Cento V., Antonello M., Colagrossi L., Merli M., Ughi N., Renica S., Matarazzo E., Di Ruscio F., Tartaglione L. (2020). Detection and quantification of SARS-CoV-2 by droplet digital PCR in real-time PCR negative nasopharyngeal swabs from suspected COVID-19 patients. PLoS ONE.

[18] Li D., Wang D., Dong J., Wang N., Huang H., Xu H., Xia C. (2020). False-Negative Results of Real-Time Reverse-Transcriptase Polymerase Chain Reaction for Severe Acute Respiratory Syndrome Coronavirus 2: Role of Deep-Learning-Based CT Diagnosis and Insights from Two Cases. Korean J. Radiol..

