# Supplementary material for: Quadruplex Droplet Digital PCR Assay for Screening and Quantification of SARS-CoV-2
Source: Int J Mol Sci. 2024 Jul 26;25(15):8157. doi: 10.3390/ijms25158157 (PMC11311395; doi:10.3390/ijms25158157)
Supplement: Supplementary file 1 [file ijms-25-08157-s001.zip › ijms-3102134-supplementary.pdf]

Supporting information

Supplementary Figure S1. Optimization of the simplex and quadruplex ddPCR assays. (a) Optimization of the ddPCR assay for the ORF1 ab target with different ratios of primers and probes. (b) Optimization of the 2-plex ddPCR assay for the ORF1 ab and RdRp targets in the FAM channel. (c) Droplet distribution in the VIC channel during the optimization of the 3-plex ddPCR assay for the ORF1 ab, RdRp, and N gene targets. (d) Droplet distribution in the VIC channel during the optimization of the 4-plex ddPCR assay. (e) Droplet distribution in the VIC channel during temperature optimization of the 4-plex ddPCR assay. (f) Droplet distribution in the FAM channel during temperature optimization of the 4-plex ddPCR assay.

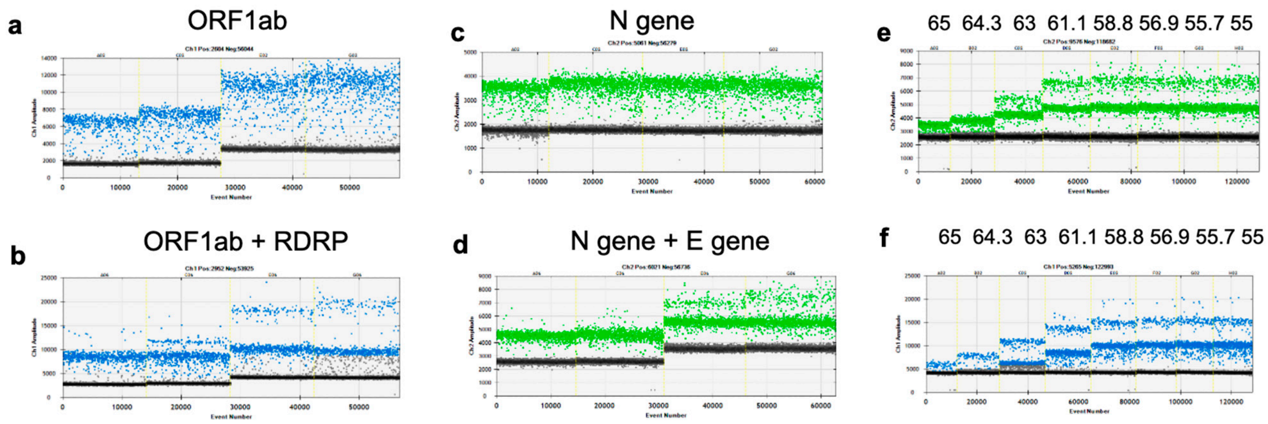

Supplementary Table S1. CRMs for specificity evaluation of ddPCR assays

Respiratory virus	Sample Name	Source
NCRM-1	2019-nCoVRNA Reference material GBW(E)091089	NCRM
RMs-2	Parainfluenza virus type 1	SIMT
RMs-3	Influenza A virus (H1 N1)	SIMT
RMs-4	Influenza B virus (Victoria)	SIMT
RMs-5	SARS-CoV	SIMT
RMs-6	MERS-CoV	SIMT

Supplementary Table S2. Primers and probes used in ddPCR analysis

Primer/probe name	Sequence (5′-3′)
SARS-CoV-2_N2-F	TTACAAACATTGGCCGCAAA
SARS-CoV-2_N2-R	GCGCGACATTCCGAAGAA
SARS-CoV-2_N2-P	VIC ACAATTTGCCCCCAGCGCTTCAG BHQ1
SARS-CoV-2_E2-F	ACAGGTACGTTAATAGTTAATAGCGT
SARS-CoV-2_E2-R	ATATTGCAGCAGTACGCACACA
SARS-CoV-2_E2-P	VIC ACACTAGCCATCCTTACTGCGCTTCG BHQ1
SARS-CoV-2_RdRP1-F	GTCATGTGTGGCGGTTCACT
SARS-CoV-2_RdRP1-R	CAACACTATTAGCATAAGCAGTTGT
SARS-CoV-2_RdRP1-P	FAM CAGGTGGAACCTCATCAGGAGATGC BHQ1
ORF1 ab-F	CCCTGTGGGTTTTACACTTAA
ORF1 ab-R	ACGATTGTGCATCAGCTGA
ORF1 ab-P	FAM CCGTCTGCGGTATGTGGAAAGGTTATGG BHQ1

Supplementary Table S3. Ct values of the practical samples in qRT-PCR assays

Practical Samples	N		ORF1 ab	
	avg ct	RSD	avg ct	RSD
S1	28.4	6.43%	29.1	5.87%
S2	31.2	5.48%	30.6	6.53%
S3	32.9	7.47%	33.2	5.93%
S4	/	/	/	/
S5	/	/	/	/
S6	/	/	/	/
S7	/	/	/	/
S8	/	/	/	/
S9	/	/	/	/
S10	/	/	/	/
S11	/	/	/	/
S12	/	/	/	/
S13	/	/	/	/
